# Association between the neutrophil-to-lymphocyte ratio and cognitive impairment: a meta-analysis of observational studies

**DOI:** 10.3389/fendo.2023.1265637

**Published:** 2023-11-28

**Authors:** Kuo-Chuan Hung, Chien-Cheng Liu, Jheng-Yan Wu, Chun-Ning Ho, Ming-Chung Lin, Chung-Hsi Hsing, I-Wen Chen

**Affiliations:** ^1^ School of Medicine, College of Medicine, National Sun Yat-sen University, Kaohsiung, Taiwan; ^2^ Department of Anesthesiology, Chi Mei Medical Center, Tainan, Taiwan; ^3^ Department of Anesthesiology, E-Da Hospital, I-Shou University, Kaohsiung, Taiwan; ^4^ Department of Nursing, College of Medicine, I-Shou University, Kaohsiung, Taiwan; ^5^ School of Medicine, I-Shou University, Kaohsiung, Taiwan; ^6^ Department of Nutrition, Chi Mei Medical Center, Tainan, Taiwan; ^7^ Department of Medical Research, Chi-Mei Medical Center, Tainan, Taiwan; ^8^ Department of Anesthesiology, Chi Mei Medical Center, Liouying, Tainan, Taiwan

**Keywords:** neutrophil-to-lymphocyte ratio, cognitive impairment, meta-analysis, age, mild cognitive impairment

## Abstract

**Background:**

Systemic inflammation is one of the underlying mechanisms of cognitive impairment. The neutrophil-to-lymphocyte ratio (NLR) has emerged as a systemic inflammation indicator. This meta-analysis aimed to evaluate the association between high NLR and cognitive impairment (CI) risk.

**Method:**

A comprehensive systematic search was conducted to identify eligible studies published until May 30, 2023. The reference group comprised patients with the lowest NLR level, whereas the exposure group comprised those with the highest NLR level. The main outcome was to examine the relationship between NLR and CI risk. The secondary outcome included the association between patient characteristics or comorbidities and CI risk.

**Results:**

This meta-analysis included 11 studies published between 2018 and 2023, involving 10,357 patients. Patients with CI had a higher NLR than those without (mean difference=0.35, 95% confidence interval [CI]: 0.26–0.44, *p* < 00001, I^2 =^ 86%). Consistently, pooled results revealed an association between high NLR and CI risk (odds ratio [OR]=2.53, 95% CI:1.67–3.82, *p*<0.0001, I^2 =^ 84%). Furthermore, aging (mean difference =4.31 years, 95% CI:2.83–5.8, *p* < 0.00001, I^2 =^ 92%), diabetes (OR=1.59, 95% CI:1.35–1.88, *p* < 0.00001, I^2 =^ 66%), and hypertension (OR=1.36, 95% CI:1.19–1.57, *p* < 0.00001, I^2 =^ 0%) were significant risk factors for CI. However, no significant associations were observed between CI and male gender (OR = 0.84, 95% CI:0.64–1.11, *p* = 0.22, I^2 =^ 81%), body mass index (mean = −0.32 kg/m^2^, 95% CI: −0.82, 0.18, *p* = 0.2, I^2 =^ 82%), alcohol consumption (OR = 1.11, 95% CI:0.95−1.3, *p* = 1.35, I^2 =^ 0%), and smoking (OR = 0.99, 95% CI:0.87–1.13, *p* = 0.86, I^2 =^ 0%). Meta-regression found that diabetes and hypertension, but not age, significantly moderated the association between NLR and CI.

**Conclusion:**

This meta-analysis showed a significant association between high NLR and increased CI risk. Moreover, meta-regression identified diabetes and hypertension, but not age, as significant moderating factors in the relationship between NLR and CI. To validate and strengthen these findings, further large-scale studies are required.

**Systematic Review Registration:**

https://www.crd.york.ac.uk/prospero/display_record.php?ID=CRD42023430384, identifier CRD42023430384.

## 1 Introduction

Mild cognitive impairment is characterized by a noticeable decline in cognitive abilities, including memory, thinking, and attention, which is greater than expected for a person’s age and education level (1). However, individuals with mild cognitive impairment can independently perform daily activities without any significant interference. Conversely, cognitive impairment is a broader term that encompasses various conditions characterized by a decline in cognitive abilities. It includes mild cognitive impairment but also extends to more severe forms of cognitive decline, including dementia. In the general population, the estimated prevalence of mild cognitive impairment is approximately 3–18.6% (2–4), whereas in stroke survivors, the prevalence may be as high as approximately 60% (5, 6). Cognitive impairment, irrespective of its degree of severity, in older adults increases susceptibility to falls, disability, and deterioration in health-related quality of life (7–9). Even mild cognitive impairment demonstrates a conversion rate to dementia ranging from 18.4% to 43% (10–12). Timely detection and treatment using both non-pharmacological and pharmacological interventions for mild cognitive impairment may delay or avert subsequent dementia development (13, 14). Moreover, the presence of preoperative cognitive impairment is associated with a substantial increase in the risk of postoperative delirium, postoperative complications, 30-day readmission, discharge to assisted care, and 1-year mortality (15). Considering the aging population, cognitive impairment has emerged as a significant global public health concern (16). Therefore, there is an urgent clinical need to identify diagnostic biomarkers that can identify older adults who are susceptible to cognitive impairment.

Evidence suggests that chronic inflammation plays a pathogenic role in the development of cognitive impairment/mild cognitive impairment and Alzheimer’s disease (AD) in older adults (17–21). For example, a study conducted over a 20-year period involving approximately 2,000 patients has provided evidence indicating a higher cognitive impairment probability in individuals exhibiting recurrent elevations or progressive increases in interleukin (IL)-6 levels (18). A previous meta-analysis showed higher peripheral levels of soluble tumor necrosis factor receptor 2, monocyte chemoattractant protein-1, and IL-6, along with decreased IL-8 levels, in patients diagnosed with mild cognitive impairment when compared with controls (22). These findings suggest an association between inflammation and cognitive decline. The neutrophil-to-lymphocyte ratio (NLR) has emerged as a preferred biomarker for assessing systemic inflammation owing to its novelty, cost-effectiveness, and suitability for extensive screening (23–25). Recently, several studies demonstrated a positive correlation between elevated NLR and cognitive impairment/mild cognitive impairment (26–28). Integrating NLR assessment into routine screening protocols may enhance the early identification of individuals at risk for cognitive decline, thereby leading to improved patient outcomes and the potential for preventive measures to mitigate cognitive impairment progression. To synthesize the clinical evidence available, the current meta-analysis and systematic review were conducted to explore the potential association between elevated NLR and increased risk of cognitive impairment in the population without previous dementia or psychiatric disease (e.g., bipolar disorder).

## 2 Methods

### 2.1 Protocol registration

This systematic review was reported in accordance with the Preferred Reporting Items for Systematic Reviews and Meta-Analyses (PRISMA) guideline (Registration number of systematic reviews in PROSPERO: CRD42023430384).

### 2.2 Eligibility criteria

Regardless of publication date or language, studies that investigated the correlation between NLR and cognitive impairment in adults were eligible. We considered both observational studies and randomized controlled trials as eligible for inclusion. Studies were excluded if they met any of the following criteria: (1) unavailability of outcomes; (2) involvement of the postoperative setting; (3) presentation as conference abstracts, case reports, duplicated studies, unpublished reports, or review articles; (4) exclusive inclusion of patients with AD (to avoid significant pre-existing cognitive impairment) or bipolar disorder (to avoid mood-related cognitive fluctuations); or (5) studies focusing on delirium or dementia as outcomes. We chose not to exclude studies centered on patients with stroke or diabetes because these diseases are recognized risk factors for cognitive impairment. Analyzing NLR in such a high-risk demographic can offer valuable perspectives.

### 2.3 Information sources and search strategies

A comprehensive systematic search was conducted across four electronic databases, including Medline (OVID), EMBASE (OVID), Google Scholar, and the Cochrane Library, to identify eligible studies published until May 30, 2023. Boolean operators (e.g., OR and AND) were employed to combine search terms in these databases. The following were the search terms used: (“NLR” or “Neutrophil*-to-lymphocyte” or “Neutrophile*-to-Lymphocyte Ratio” or “Neutrophil*/lymphocyte” or “Neutrophil* to lymphocyte ratio” or “Neutrophil* lymphocyte ratio”) and (“Cognitive impairment” or “Impaired cognition” or “Cognitive decline” or “Cognitive dysfunction” or “Cognitive disability” or “Cognitive disorder” or “Cognitive impairment” or “Cognitive deficit”).

To ensure an exhaustive search, controlled vocabulary terms (e.g., MeSH terms) were employed as search terms. Moreover, to identify studies that met the eligibility criteria, reference lists of pertinent articles, including review articles, were scrutinized. The relevant search strategies for one of the databases (e.g., Medline [OVID]) are presented in **Supplemental Table 1**.

### 2.4 Selection process and data collection

The study eligibility determination process included the following three steps: (1) duplicated articles were excluded using EndNote software; (2) two authors independently screened titles and abstracts to identify eligible articles for full-text review; and (3) studies meeting the inclusion criteria following full-text reading were included. Any discrepancies encountered during the selection process were resolved by consultation with a third author.

Data obtained from each study encompassed the following information: study population, first author’s name, publication year, age, sex, body mass index (BMI), method of diagnosing cognitive impairment, sample size, NLR value, type of odds ratio (OR) (e.g., adjusted or non-adjusted), and country. In cases where discrepancies arose, resolution was sought through consultation with a third author. Furthermore, in instances where certain information was absent from the article, attempts were made to contact the corresponding author on three separate occasions to acquire the missing data.

### 2.5 Definitions and outcomes

In the present meta-analysis, the term “cognitive impairment” also included mild cognitive impairment. The main objective of this study was to examine the relationship between NLR and cognitive impairment risk. The primary outcome measure used for analysis was OR, which assessed the association between NLR and the likelihood of developing cognitive impairment. In the present meta-analysis, the reference group comprised of patients with the lowest NLR, whereas the exposure group comprised of those with the highest NLR. The NLR cutoff values or diagnosis of cognitive impairment were based on the criteria used in each individual study, rather than applying a single unified criterion. The secondary outcomes included the association between patient characteristics, comorbidities, and cognitive impairment risk.

### 2.6 Quality assessment

The Newcastle-Ottawa Scale (NOS) was used to evaluate the methodological rigor of the studies included in the analysis. The assessment encompassed three key aspects: subject selection, comparability of groups, and outcome or exposure assessment. Each study was assigned a quality grade based on a scale ranging from low (0–3), moderate (4–6), to high (7–9). Any discrepancies that arose during the evaluation process were resolved through consensus.

### 2.7 Statistical analyses

The Cochrane Review Manager (RevMan 5.3; Copenhagen: The Nordic Cochrane Center, The Cochrane Collaboration, 2014) and comprehensive Meta-Analysis (CMA) V3 software (Biostat, Englewood, NJ, USA) was used for data synthesis. To account for potential heterogeneities arising from variations in clinical settings, we employed the Mantel–Haenszel random-effects model to analyze dichotomous outcome data, presenting the results as ORs accompanied by 95% confidence intervals (CIs). The mean differences (MDs) and their corresponding 95% CIs were reported for outcomes involving continuous variables. Significant heterogeneity was defined as an I^2^ value > 50%. Through visual examination of a funnel plot, publication bias was assessed for the outcomes mentioned in at least ten studies. Furthermore, the potential influence of individual studies on the overall results was assessed using a “leave-one-out” sensitivity analysis. To investigate the possible moderating influences of risk factors on the relationship between NLR and cognitive impairment, we performed meta-regression analyses. A significant coefficient implies that the moderator variable significantly influences the strength of the association between NLR and cognitive impairment. All comparisons were subjected to two-tailed tests, with statistical significance set at a *p*-value < 0.05.

## 3 Results

### 3.1 Search results and study characteristics

**Figure 1:**
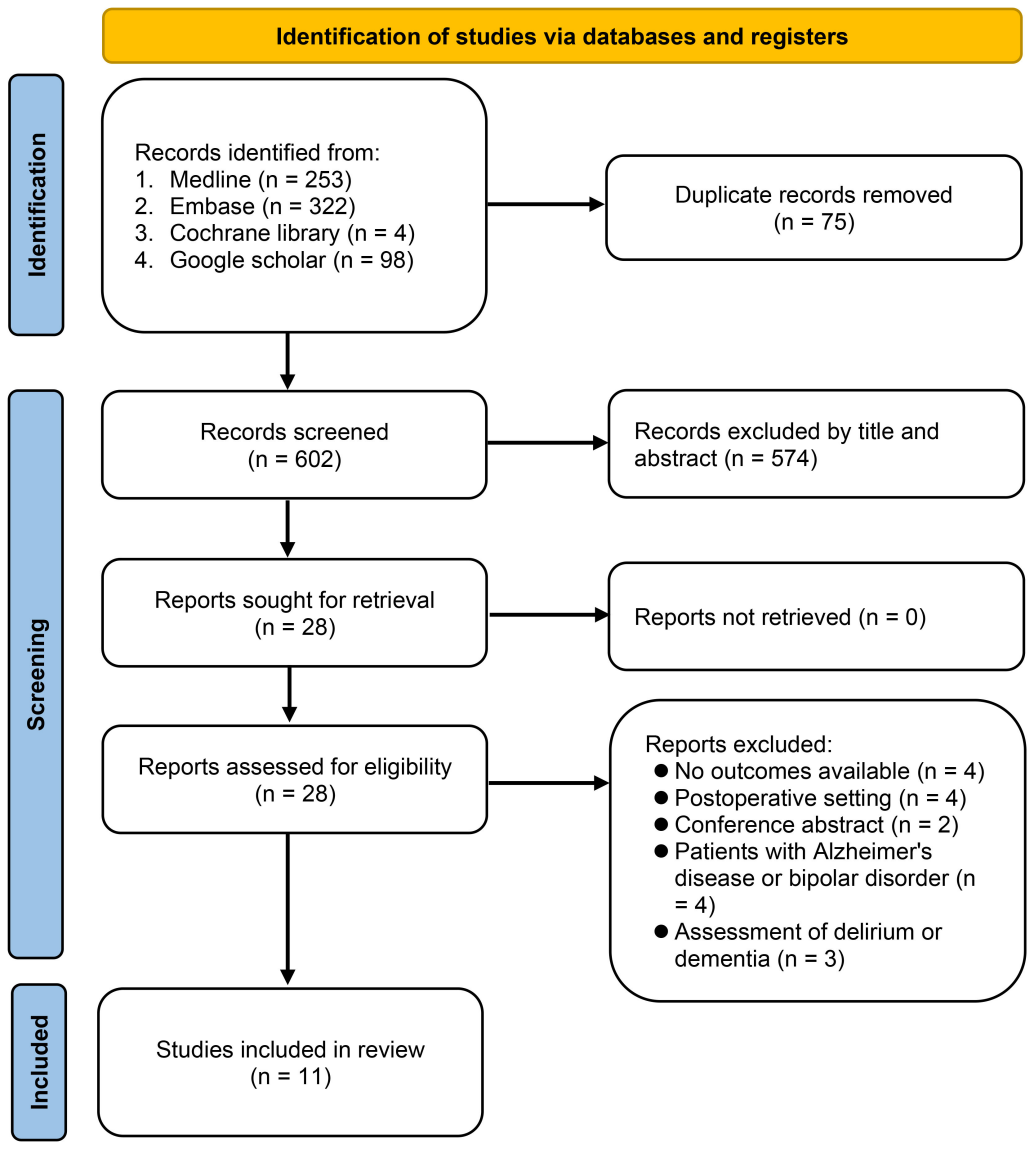
Flow chart for study selection.

The study selection procedure is illustrated in **Figure 1**. Initially, 677 potentially pertinent articles were retrieved from the electronic databases. Among these, 75 duplicates were eliminated and an additional 574 articles were deemed unsuitable based on their titles and abstracts. Subsequently, to assess potential eligibility, the remaining 28 studies were subjected to a comprehensive full-text review. After applying the exclusion criteria, 17 studies were excluded, leaving 11 studies published between 2018 and 2023 (26–36). These studies involved 10,357 patients and were included in the meta-analysis. Of the articles selected for this meta-analysis, all were identified as observational studies.

The characteristics of the 11 included studies are summarized in **Table 1**. The analysis included four studies involving patients aged ≥ 60 years or older (26, 28, 30, 36), four studies involving patients with stroke (27, 29, 33, 35), two studies focused on patients with type 2 DM (32, 34), and one study involving patients with metabolic syndrome (31). In one study (36), even though the main emphasis was on the relationship between NLR and delirium risk, it also presented valuable data on the association between NLR and cognitive impairment. For our analysis, we extracted and incorporated only the data pertaining to the link between NLR and cognitive impairment, ensuring it met our inclusion criteria. All included studies enrolled participants of both sexes, with the percentage of males ranging from 38.9% to 76.1%. The mean or median age of recruited individuals varied across studies, ranging from 47.9 to 84 years. Regarding the sample size, the number of patients included in the studies varied, ranging from 60 to 4,579. Among them, two studies were large-scale studies, including 2,479 (30) and 4,579 (28) patients, respectively, whereas two studies were small-scale studies, involving only 60 patients (31, 32). Ten studies explicitly stated the diagnostic method used for cognitive impairment (26–35), whereas one study did not provide any description (36). Among the included studies, nine reported ORs, with seven (26–30, 33, 34) and two (32, 36) studies presenting adjusted and unadjusted ORs, respectively. Additionally, two studies exclusively provided NLR values for patients with and without cognitive impairment (31, 35). The studies were conducted across multiple countries, including China (26, 28–30, 33–36), South Korea (27), Thailand (31), and India (32), with China being the predominant country of study.

**Table 1:** Characteristics of studies (n = 11).

Studies	Population	Age (years) ¶	Male (%) ¶	N	Diagnosis for CI	Adjusted OR (Y/N)	High NLR	Low NLR	Country	NOS
An 2019 (26)	Adults aged 65 or older	73.1 vs. 71.1	42 vs. 47	339	MPC	Y	>2.07	<2.07	China	9
Hou 2022 (27)	Adults with cerebral small vessel disease	61.2 vs. 64.5	43.2 vs. 57.5	147	MoCA score	Y	>1.89	<1.89	China	9
Lee 2021 (28)	Adults with ischemic stroke	66.7 vs. 62	53.5 vs. 67.2	345	K-VCIHS-NP	Y	≥3.8	≤1.57	Korea	9
Li 2023 (29)	Adults aged 60 years or older	73.5 vs. 68.6	46.5 vs. 46.5	2479	CCSZ	Y	>2.88	≤1.53	China	7
Liu 2020 (30)	Adults aged 60 or older	67.6‡	52‡	4579	AMT	Y	>2.64	<1.53	China	9
Pipatpiboon 2022 (31)	Adults with metabolic syndrome	57.47‡	47‡	60	MOCA score	NA	>1.84	<1.61	Thailand	6
Sasirekha 2018 (32)	Adults with type II DM	50.8 vs. 47.9	38.9 vs. 54.8	60	MMSE	N	>2	<2	India	6
Shang 2022 (33)	Adults with mild acute ischemia stroke	62 vs. 61	68.4 vs. 76.1	454	MOCA score	Y	≥4.05	≤1.62	China	8
Yu 2023 (34)	Adults with type 2 DM	60 vs. 55	45.2 vs. 49.4	787	MOCA score	Y	>1.99	<1.48	China	8
Zha 2022 (35)	Adults with ischemia stroke	67.3 vs. 60.1	42.5 vs. 67.1	367	MMSE	NA	>2.73	<2.14	China	6
Zhao 2021 (36)	Hospitalized patients aged ≥ 70 years	84‡	71.20‡	740	NA	N	>3.63	≤3.63	China	6

MPC, modified Petersen’s criteria; MoCA, Montreal Cognitive Assessment; K-VCIHS-NP, Korean version of the Vascular Cognitive Impairment

Harmonization Standards-Neuropsychological Protocol; AMT, Abbreviated Mental Test; MMSE, Mini‐Mental State Examination; CCSZ, Composite cognitive score Z-score; OR, odd ratio; Y, yes; N, no; NA, not available; CI, cognitive impairment; ¶present as patients with cognitive impairment vs. without cognitive impairment; ‡overall population; NOS, Newcastle-Ottawa Scale; NLR, neutrophil-to-lymphocyte ratio

The quality of the studies is summarized in **Table 1**. Four of the 11 studies evaluated were considered to have potential biases (e.g., NOS = 6), indicating some limitations or weaknesses in their study design. Conversely, seven studies were considered to have a low risk of bias (e.g., NOS range:7–9), suggesting that they have a more robust and reliable methodology.

### 3.2 Outcomes

#### 3.2.1 Risk of cognitive impairment in patients with high NLR ratio

**Figure 2:**
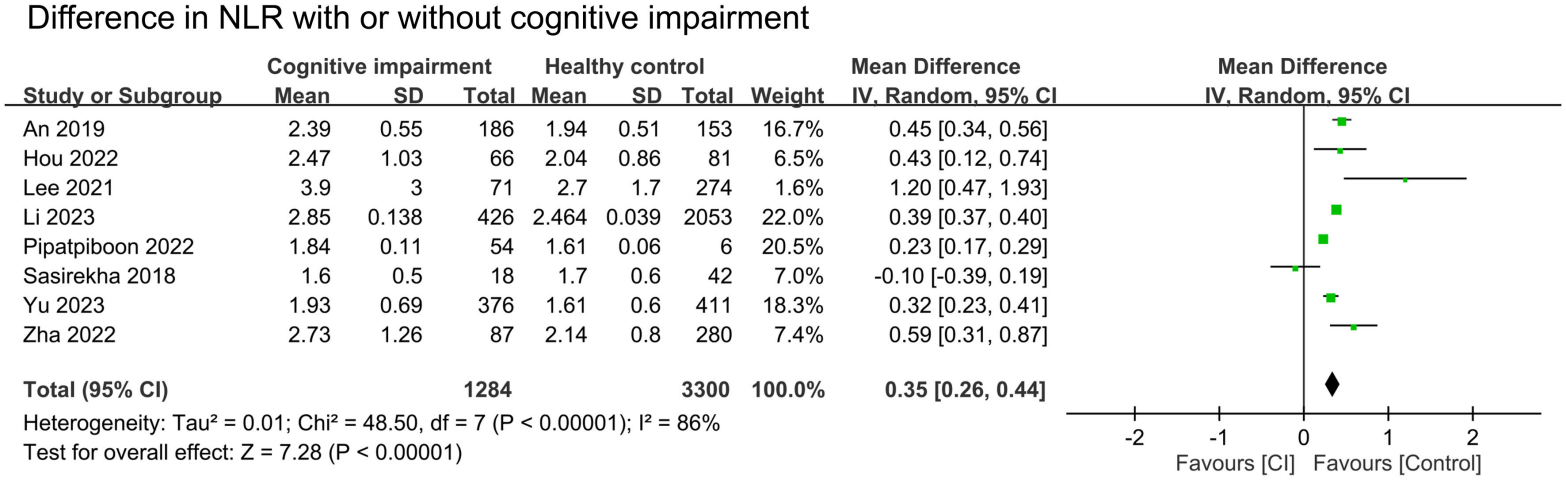
Forest plot comparing the neutrophil-to-lymphocyte ratio (NLR) in patients with cognitive impairment (CI) and those without. The point estimates and their 95% confidence intervals are represented by squares and horizontal lines, respectively. A point estimate to the right of the centerline indicates a higher NLR in the CI group relative to the control group, denoted by ‘favours [Control]’.

**Figure 3:**
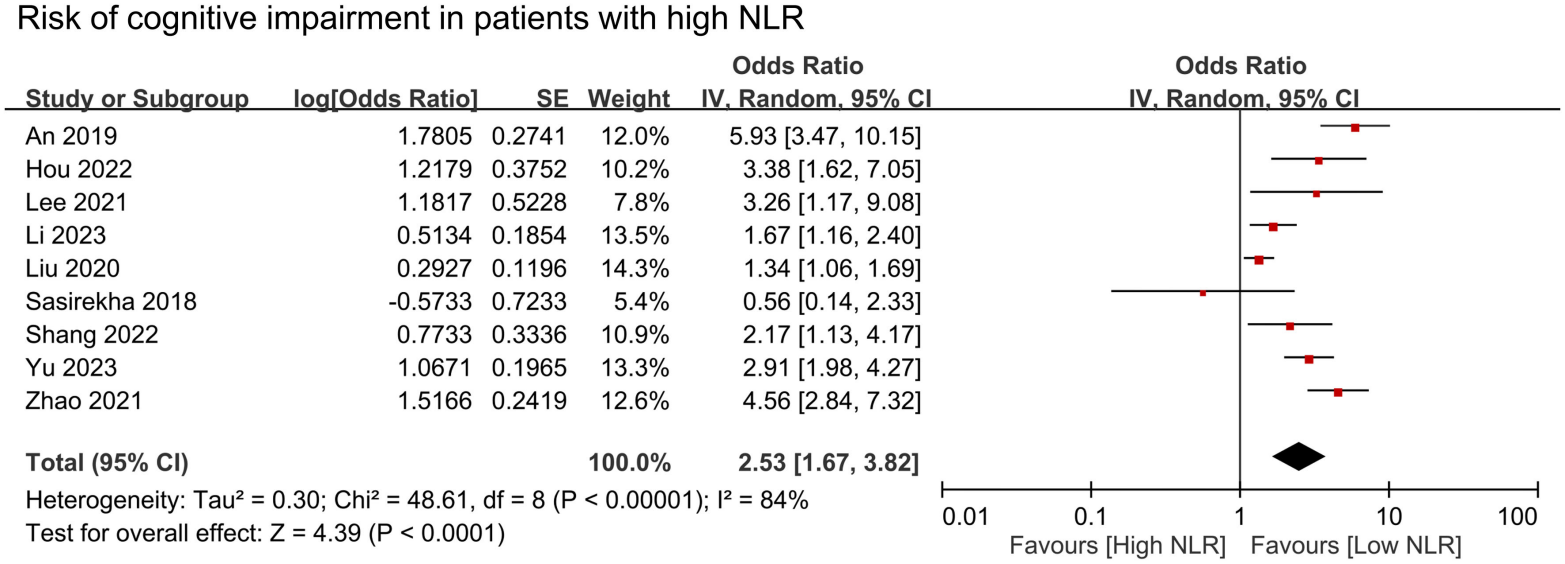
Forest plot showing cognitive impairment risk in patients with high neutrophil-to-lymphocyte ratio (NLR). IV, inverse variation; CI, confidence interval; SE, standard error. The point estimates and their 95% confidence intervals are represented by squares and horizontal lines, respectively. A point estimate to the right of the centerline indicates a higher cognitive impairment risk in the high NLR group relative to the low NLR group, denoted by ‘favours [low NLR]’.

The results of pooled data from included studies showed significantly higher NLR in patients with cognitive impairment (MD = 0.35, 95% CI:0.26–0.44, *p* < 00001, I^2 =^ 86%, sensitivity analysis: consistent, range of MD: 0.33-0.38, all p value<0.05) (**Figure 2**) (26, 27, 29–32, 34, 35). Nine studies provided information to estimate the pooled risk of cognitive impairment in patients with a high NLR (primary outcome). Meta-analysis showed an association between a high NLR and cognitive impairment risk (OR = 2.53, 95% CI:1.67–3.82, *p* < 0.0001, I^2 =^ 84%) (**Figure 3**) (26–30, 32–34, 36). Sensitivity analysis showed consistent findings (range of OR: 2.25-2.84, all *p* value<0.05) when one study was removed individually.

#### 3.2.2 Other risk factors for cognitive impairment

**Figure 4:**
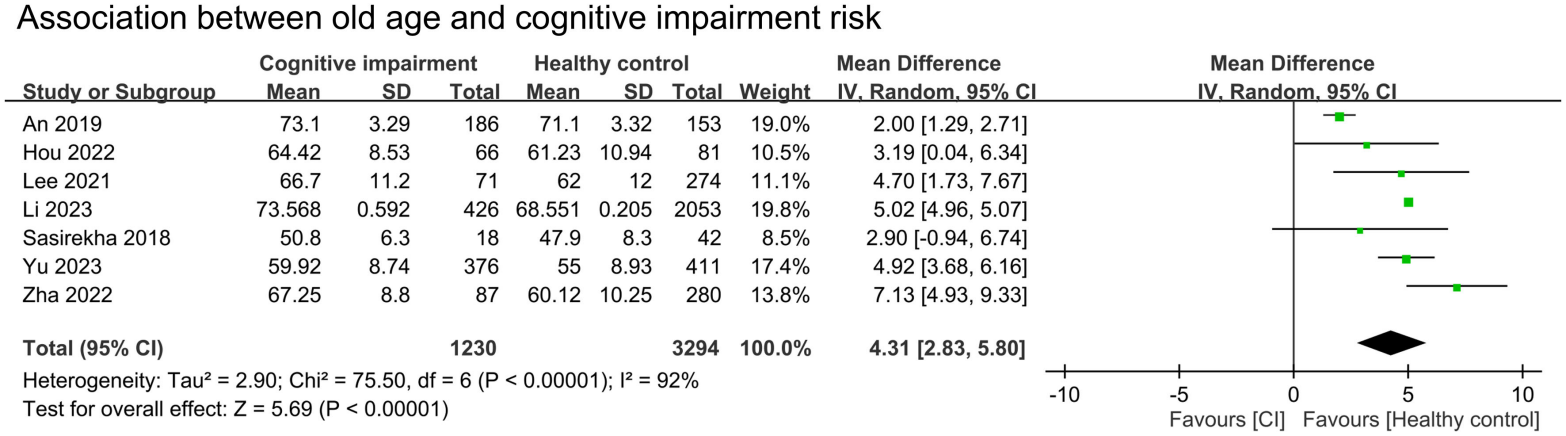
Forest plot showing an association between old age and cognitive impairment risk. IV, inverse variation; CI, confidence interval. The point estimates and their 95% confidence intervals are represented by squares and horizontal lines, respectively. A point estimate to the right of the centerline suggests a younger age in the healthy control group relative to the cognitive impairment group, denoted by ‘favours [healthy control]’.

**Figure 5:**
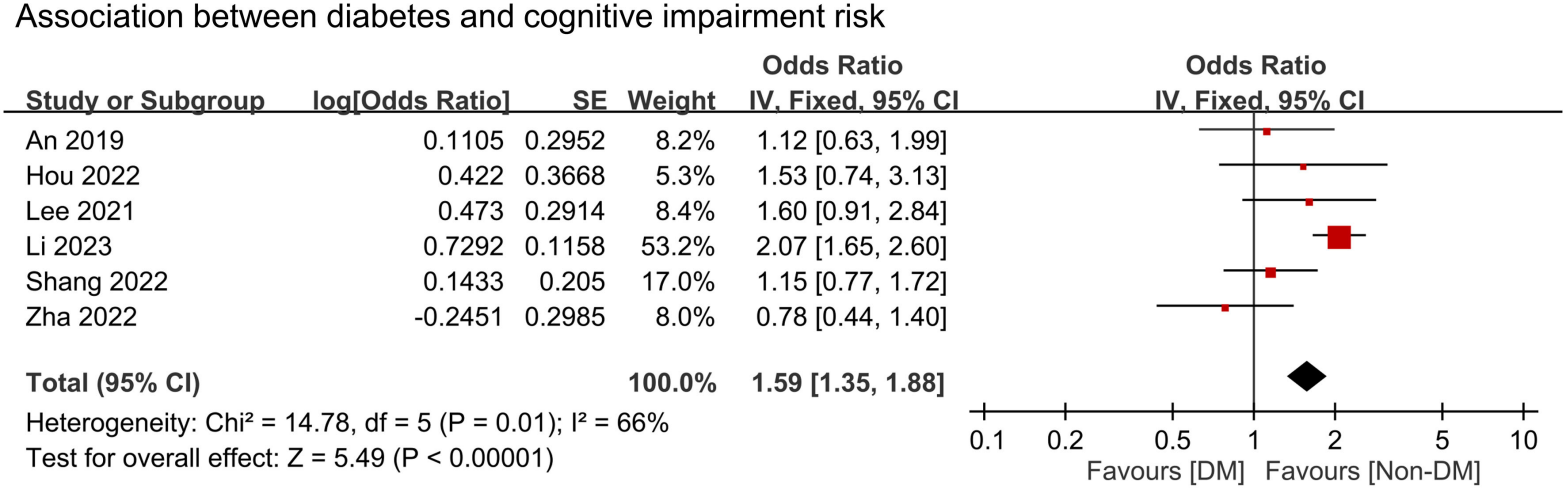
Forest plot showing an association between diabetes and cognitive impairment risk. IV, inverse variation; CI, confidence interval. The point estimates and their 95% confidence intervals are represented by squares and horizontal lines, respectively. A point estimate to the right of the centerline suggests a higher cognitive impairment risk in the DM group relative to the Non-DM group, denoted by ‘favours [Non-DM]’.

**Figure 6:**
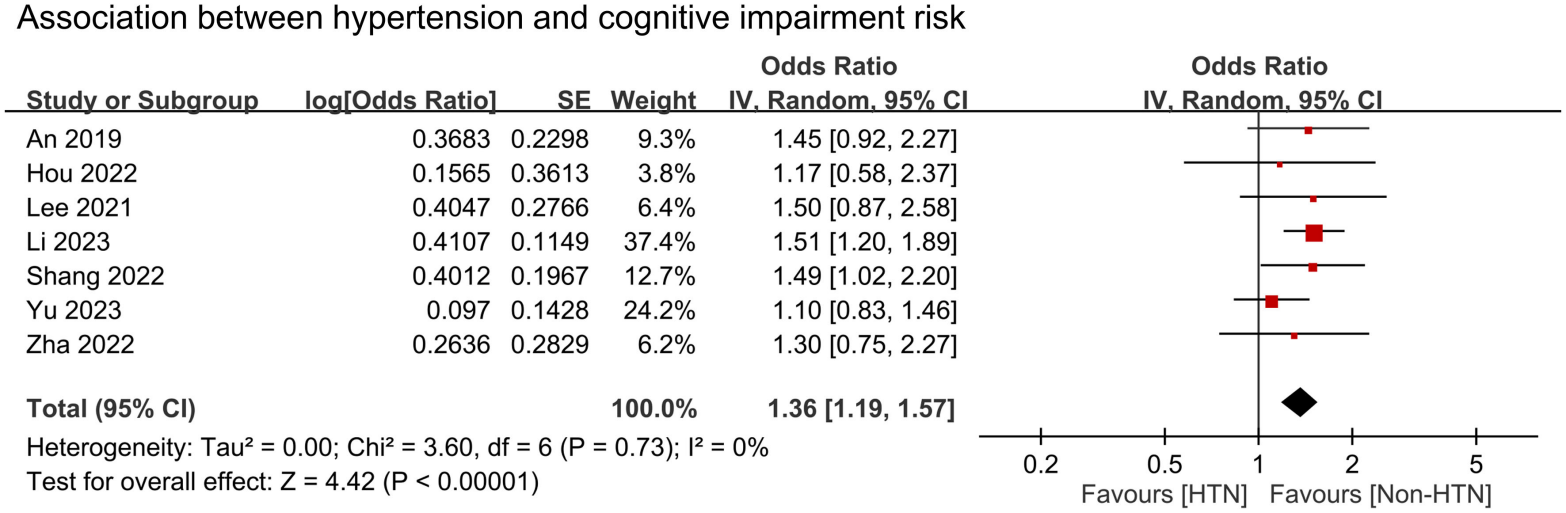
Forest plot showing an association between hypertension and cognitive impairment risk. IV, inverse variation; CI, confidence interval. The point estimates and their 95% confidence intervals are represented by squares and horizontal lines, respectively. A point estimate to the right of the centerline suggests a higher cognitive impairment risk in the HTN group relative to the Non-HTN group, denoted by ‘favours [Non-HTN]’.

We conducted a meta-analysis to examine the association between cognitive impairment and various factors. Results indicated a significant association between cognitive impairment and old age (MD = 4.31 years, 95% CI:2.83–5.8, *p* < 0.00001, I^2 =^ 92%, sensitivity analysis: consistent, range of MD: 3.86-5.02, all p value<0.05) (**Figure 4**), diabetes (OR = 1.59, 95% CI:1.35–1.88, *p* < 0.00001, I^2 =^ 66%, sensitivity analysis: inconsistent, range of OR: 1.18-1.7) (**Figure 5**), and hypertension (OR = 1.36, 95% CI:1.19–1.57, *p* < 0.00001, I^2 =^ 0%, sensitivity analysis: consistent, range of OR: 1.29-1.46, all p value<0.05) (**Figure 6**). However, no association was observed between cognitive impairment and male sex (OR = 0.84, 95% CI:0.64–1.11, *p* = 0.22, I^2 =^ 81%, sensitivity analysis: consistent, range of OR: 0.77-0.95, all p value>0.05) (**Supplemental Figure 1**), BMI (MD = −0.32 kg/m^2^, 95% CI: −0.82, 0.18, *p* = 0.2, I^2 =^ 82%, sensitivity analysis: inconsistent, range of MD: -0.16 to -0.52) (**Supplemental Figure 2**), alcohol consumption (OR = 1.11, 95% CI:0.95−1.3, *p* = 1.35, I^2 =^ 0%, sensitivity analysis: consistent, range of OR: 1.03-1.17, all p value>0.05)(**Supplemental Figure 3**), and smoking (OR = 0.99, 95% CI:0.87–1.13, *p* = 0.86, I^2 =^ 0%, sensitivity analysis: consistent, range of OR: 0.92-1.02, all p value>0.05) (**Supplemental Figure 4**). Owing to the limited number of studies available for each outcome, the funnel plots were not examined.

#### 3.2.3 Meta-regression

**Figure 7:**
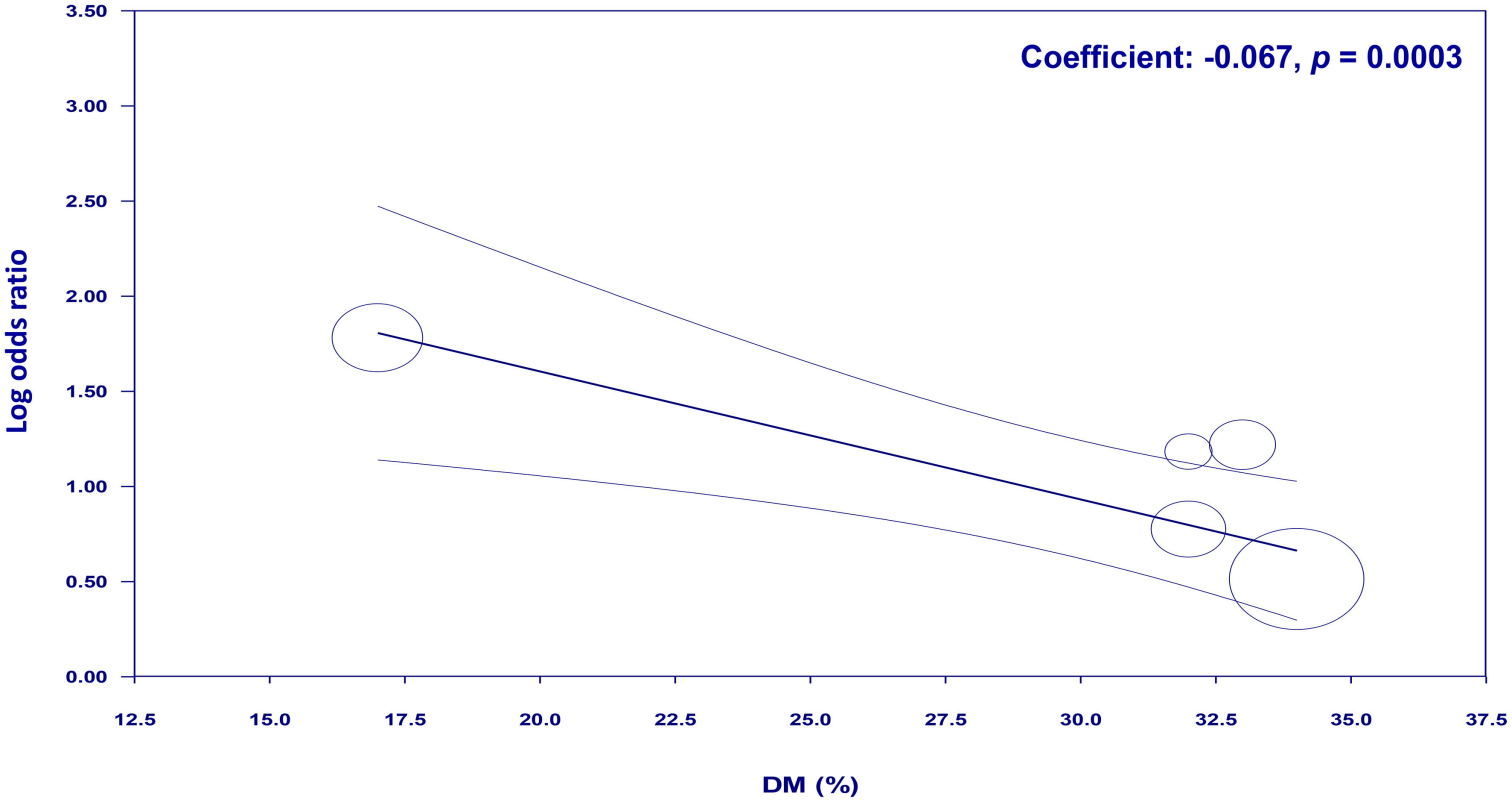
Meta-regression showing the potential moderating effect of diabetes on the association between neutrophil-to-lymphocyte ratio (NLR) and cognitive impairment.

**Figure 8:**
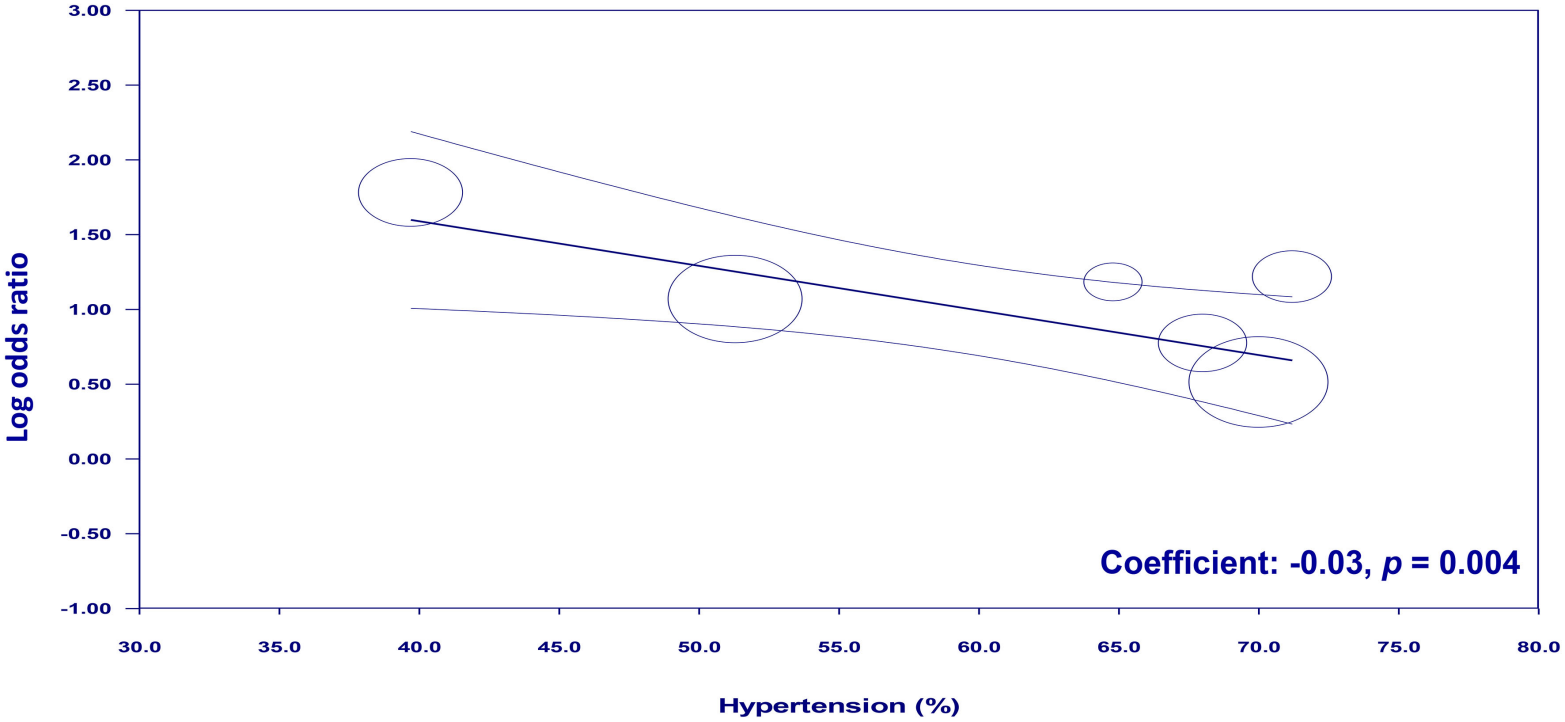
Meta-regression showing the potential moderating effect of hypertension on the association between neutrophil-to-lymphocyte ratio (NLR) and cognitive impairment.

**Figure 9:**
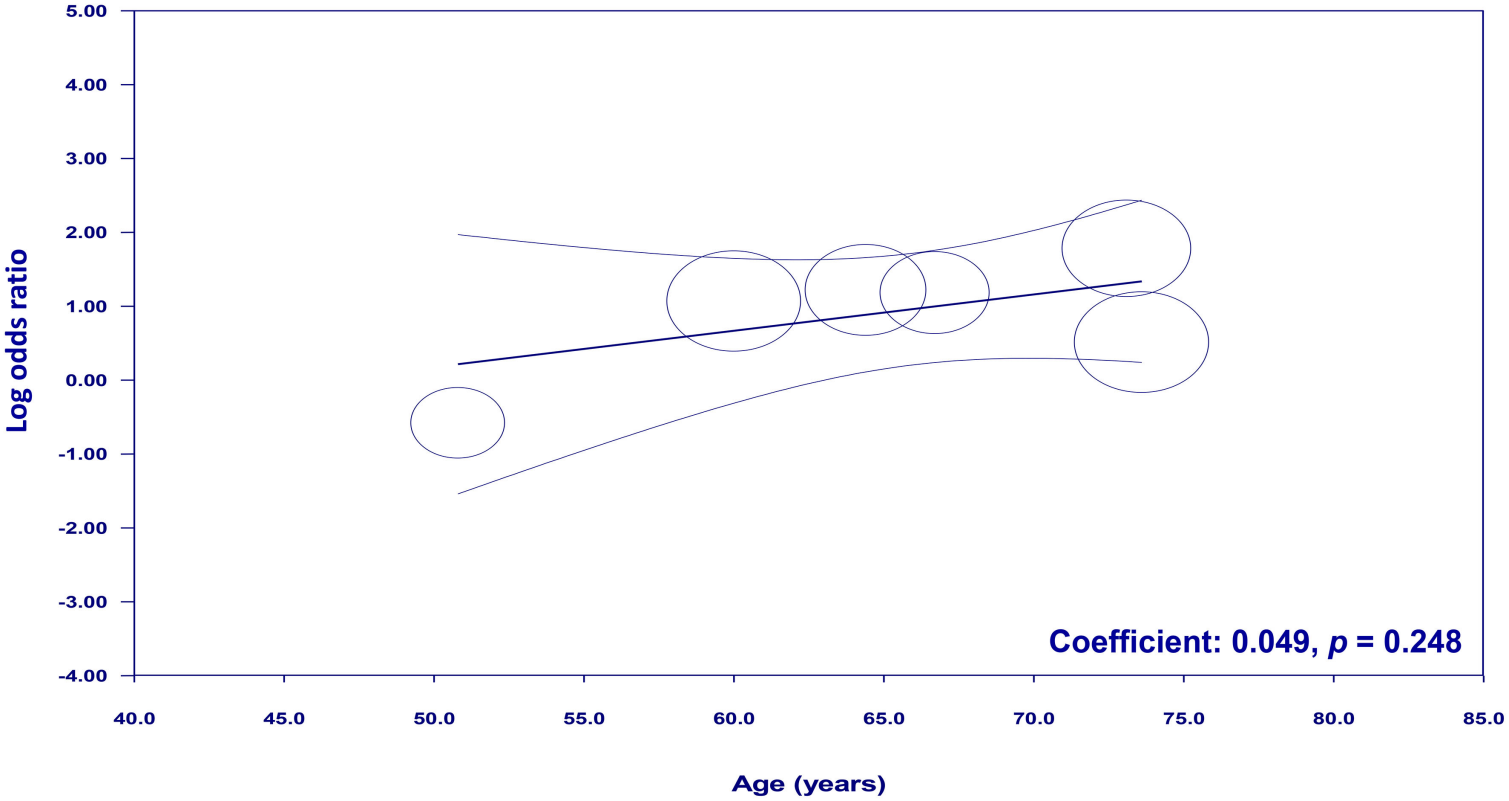
Meta-regression showing no moderating effect of age on the association between neutrophil-to-lymphocyte ratio (NLR) and cognitive impairment.

To examine the potential moderating effects of age, diabetes, and hypertension on the association between NLR and cognitive impairment, meta-regression analyses were conducted. The mean age of participants, proportion of patients with diabetes, and proportion of patients with hypertension were entered as moderators in separate univariate meta-regression models. These findings reveal that both diabetes (coefficient: -0.067, *p* = 0.0003) (**Figure 7**) and hypertension (coefficient: -0.03, *p* = 0.004) (**Figure 8**) play a significant role as covariates in the relationship between NLR and cognitive impairment. On the other hand, age doesn’t possess a comparable covariate linkage in this setting (coefficient: 0.049, *p* = 0.248) (**Figure 9**).

## 4 Discussion

This meta-analysis included 11 studies published between 2018 and 2023, involving 10,357 patients. Pooled results revealed that patients with cognitive impairment had a higher NLR than those without cognitive impairment. From the nine studies that provided relevant data, a meta-analysis was conducted to estimate the pooled risk of cognitive impairment in patients with a high NLR, which showed a significant association (OR = 2.53). Our meta-regression indicates diabetes and hypertension, but not age, as significant moderating factors influencing the association between NLR and risk of cognitive impairment.

Systemic inflammation has emerged as a significant contributor to cognitive impairment (37–39). In an animal study, aging mice were found to be more prone to episodic systemic inflammation, and cognitive impairments in these mice were linked to increased oxidative stress and abnormal cytokine production in the brain, emphasizing the significance of neuroinflammation and oxidative stress in age-related cognitive decline (40). Several studies have also demonstrated an association between elevated levels of proinflammatory markers, including C-reactive protein, IL-6, and tumor necrosis factor-alpha (TNF-α), and cognitive decline as well as an increased risk of neurodegenerative disorders (e.g., AD and vascular dementia) (41–44). For example, a previous study of 300 community-dwelling participants with mild-to-severe AD reported that acute systemic inflammatory events were associated with a two-fold increase in cognitive decline over a 6-month period, whereas high TNF-α baseline levels were associated with a four-fold increase in cognitive decline (37). The negative impact of systemic inflammation on cognitive function is likely due to several factors, including the ability of chronic inflammation to cause vascular dysfunction, which can result in impaired cerebral blood flow and oxygenation, ultimately compromising neuronal function and cognitive processes (45, 46). Furthermore, inflammatory mediators may directly affect the brain by disrupting the blood–brain barrier, activating microglia, and releasing neurotoxic substances, ultimately leading to neuroinflammation and neuronal damage (41).

Detecting cognitive impairment early and potentially preventing its progression can significantly improve the quality of life of older adults while also reducing the social, psychological, and economic burdens faced by their families and caregivers. In the present meta-analysis, nine studies were analyzed and showed a significant association (OR = 2.53) between high NLR and cognitive impairment risk, which was consistent even after sensitivity analyses. The findings of this meta-analysis provide robust evidence to support the notion that a high NLR is associated with an increased risk of cognitive impairment. Notably, the high heterogeneity observed among the included studies highlights the necessity for additional research to investigate optimal NLR threshold values for cognitive impairment detection. Nevertheless, our research findings provide evidence supporting the clinical applicability of the NLR as a promising biomarker. Integrating NLR assessment into routine screening protocols may enhance the early identification of individuals at risk for cognitive decline, thereby leading to improved patient outcomes and the potential for preventive measures to mitigate cognitive impairment progression.

Our findings indicate a significant association between cognitive impairment and factors including advanced age, diabetes, and hypertension, consistent with the existing literature on the subject. For example, a previous meta-analysis of 19 studies and a total of 44,714 participants reported that individuals with diabetes had a significantly increased risk of mild cognitive impairment (relative risk:1.21) compared to those without diabetes (47). Furthermore, a meta-analysis involving 112,632 community-dwelling Chinese populations aged > 55 years reported an increase in the prevalence of mild cognitive impairment with advancing age (48). A prospective community-based cohort study involving 918 individuals reported an association between hypertension and a higher risk of developing mild cognitive impairment (hazard ratio,1.40) following a mean follow-up period of 4.7 years (49). Meta-regression analysis revealed that diabetes and hypertension, rather than age, played significant roles as moderating factors influencing the relationship between the NLR and the risk of cognitive impairment. As a result, future studies seeking to employ NLR as a screening tool for cognitive impairment should carefully consider the impact of these modulators.

No associations between cognitive impairment and male sex, BMI, alcohol consumption, or smoking were observed in this study. A previous study that analyzed data from three large clinical trials observed that participants classified as obese (BMI ≥ 30 kg/m^2^) had a 29% higher prevalence of cognitive impairment than those with normal weight to overweight (50). Moreover, obesity was linked to a 1.29-fold higher prevalence of cognitive impairment even after adjusting for age, sex, diabetes, and hypertension (50). In the current meta-analysis, no significant relationship between cognitive impairment and BMI was noted. This lack of correlation may be attributed to the inclusion of participants who were not obese (e.g., mean BMI < 30 kg/m^2^ in all individuals). Conflicting findings have been reported in the current literature regarding cigarette smoking and cognitive impairment. During a 10-year follow-up period of 1,436 participants without cognitive impairment at baseline, both past and current smokers had a reduced likelihood of developing cognitive impairment compared with those who had never smoked, indicating a potential protective effect of smoking on cognitive function (51). In contrast, a study involving 3,012 participants aged 60 years reported that current smoking and alcohol consumption are significantly associated with a higher risk of cognitive impairment risk (52). Further studies are needed to elucidate these associations and better understand their underlying mechanisms.

This study has some limitations. First, the included studies were restricted to specific populations, including patients with ischemic stroke, which may restrict the generalizability of the findings to a broader population. Second, small sample sizes may increase the risk of bias and compromise the statistical power required to accurately detect small associations. Third, discrepancies were observed in the cognitive diagnosis criteria employed across studies, leading to heterogeneity and potentially impacting the comparability of findings. Additionally, the extent of adjustment for confounding factors varied among studies, with some performing adjusted analyses and others omitting this crucial step. This variability may have influenced the interpretation of the results and the ability to adequately control for potential confounders. Finally, most studies were conducted in China. Consequently, caution should be exercised when attempting to generalize these findings to other geographical regions.

## 5 Conclusion

This meta-analysis showed a higher NLR in patients with cognitive impairment, indicating a potential association between the NLR and cognitive decline. Moreover, high NLR was significantly associated with an increased risk of cognitive impairment, with an OR of 2.53. Meta-regression showed that diabetes and hypertension were identified as significant moderating factors in the relationship between NLR and CI, while age did not show a significant moderating effect. Further large-scale investigations with diverse populations and standardized diagnostic criteria are warranted to validate and strengthen our findings.

## Data availability statement

The original contributions presented in the study are included in the article/**Supplementary Material**, further inquiries can be directed to the corresponding author/s.

## Author contributions

KH: Conceptualization, Data curation, Writing – original draft. CL: Investigation, Methodology, Software, Writing – original draft. JW: Formal Analysis, Software, Validation, Writing – original draft. CH: Investigation, Project administration, Resources, Writing – original draft. ML: Project administration, Resources, Validation, Writing – original draft. CH: Investigation, Methodology, Validation, Writing – original draft. IC: Supervision, Writing – original draft, Writing – review & editing.
