# Supplementary material for: Association between the neutrophil-to-lymphocyte ratio and cognitive impairment: a meta-analysis of observational studies
Source: Front Endocrinol (Lausanne). 2023 Nov 28;14:1265637. doi: 10.3389/fendo.2023.1265637 (PMC10715314; doi:10.3389/fendo.2023.1265637)

**Supplemental Table 1.** Search strategy for Medline

| 1 | ("NLR" or "Neutrophil*-to-lymphocyte" or "Neutrophile*-to-Lymphocyte Ratio" or "Neutrophil*/lymphocyte" or "Neutrophil* to lymphocyte ratio" or "Neutrophil* lymphocyte ratio").mp. |
| --- | --- |
| 2 | ("Cognitive impairment" or "Impaired cognition" or "Cognitive decline" or "Cognitive dysfunction" or "Cognitive disability" or "Cognitive disorder" or "Cognitive impairment" or "Cognitive deficit").mp. |
| 3 | exp "Cognitive Dysfunction"/ |
| 4 | 1 and (2 or 3) |

**Supplemental Figure 1.** Forest plot showing no relationship between male gender and cognitive impairment risk. IV: inverse variation; CI: confidence interval


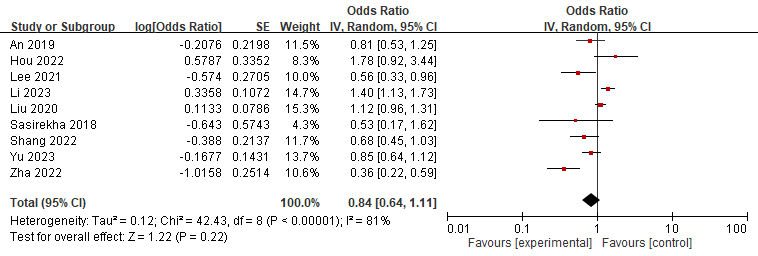


**Supplemental Figure 2.** Forest plot showing no relationship between body mass index (BMI) and cognitive impairment risk. IV: inverse variation; CI: confidence interval


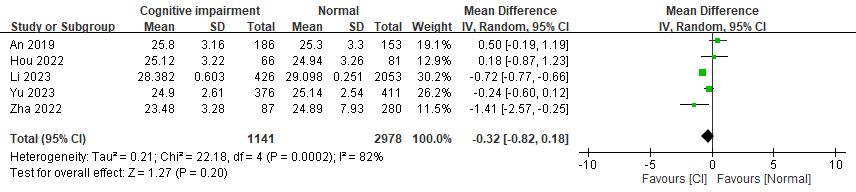


**Supplemental Figure 3.** Forest plot showing no relationship between drinking history and cognitive impairment risk. IV: inverse variation; CI: confidence interval


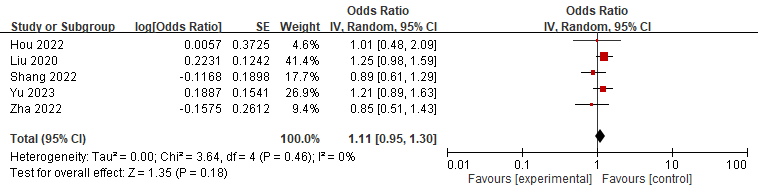


**Supplemental Figure 4.** Forest plot showing no relationship between smoking history and cognitive impairment risk. IV: inverse variation; CI: confidence interval


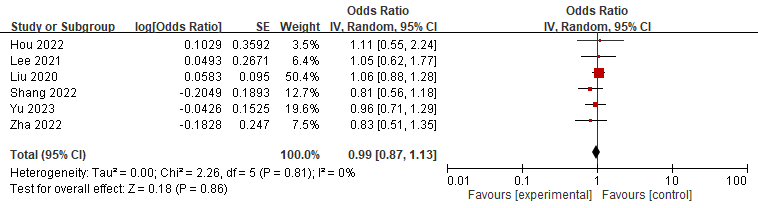

Supplement: Supplementary file 1 [file DataSheet_1.docx]
